# Transgenic *Chlamydomonas* Expressing Human Transient Receptor Potential Ankyrin 1 (TRPA1) Channels to Assess the Effect of Agonists and Antagonists

**DOI:** 10.3389/fphar.2020.578955

**Published:** 2020-09-29

**Authors:** Megumi Yoshida, Ryodai Yamamiya, Yuto Shimizu, Kenjiro Yoshimura

**Affiliations:** ^1^ Department of Machinery and Control Systems, College of Systems Engineering and Science, Shibaura Institute of Technology, Saitama, Japan; ^2^ Bio-Inteligence for Well Being, Shibaura Institute of Technology, Saitama, Japan

**Keywords:** TRP channel, *Chlamydomonas reinhardtii*, TRPA1, phototaxis, bioassay, drug development, cell motility, cilia and flagella

## Abstract

Transient receptor potential ankyrin 1 (TRPA1) channel is an ion channel whose gating is controlled by agonists, such as allyl isothiocyanate (AITC), and temperature. Since TRPA1 is associated with various disease symptoms and chemotherapeutic side effects, it is a frequent target of drug development. To facilitate the screening of TRPA1 agonists and antagonists, this study aimed to develop a simple bioassay for TRPA1 activity. To this end, transgenic *Chlamydomonas reinhardtii* expressing human TRPA1 was constructed. The transformants exhibited positive phototaxis at high temperatures (≥20°C) but negative phototaxis at low temperatures (≤15°C); wild-type cells showed positive phototaxis at all temperatures examined. In the transgenic cells, negative phototaxis was inhibited by TRPA1 antagonists, such as HC030031, A-967079, and AP18, at low temperatures. Negative phototaxis was induced by TRPA1 agonists, such as icilin and AITC, at high temperatures. The effects of these agonists were blocked by TRPA1 antagonists. In wild-type cells, none of these substances had any effects on phototaxis. These results indicate that the action of TRPA1 agonists and antagonists can be readily assessed using the behavior of *C. reinhardtii* expressing human TRPA1 as an assessment tool.

## Introduction

Living organisms have developed sensors for detecting environmental conditions and the status of homeostasis. Transient receptor potential (TRP) channels compose a large superfamily of ion channels that respond to chemical, thermal, and mechanical stimuli (Clapham, 2003). Mammalian TRP channels are classified into six families: TRPA, TRPC, TRPM, TRPML, TRPP, and TRPV, which can be further classified into subfamilies, such as TRPV1 and TRPV2. For example, TRPV1 is activated by both noxious heat and pungent substances such as capsaicin (a component of chili peppers) (Caterina et al., 1997). TRPA1 responds to both temperature and various agonists, including allyl isothiocyanate (AITC, a component of wasabi) and bradykinin (Story et al., 2003; Bandell et al., 2004; Jordt et al., 2004). In addition, TRP channels help regulate the generation of action potentials in nerve cells and control various functions in nonexcitable cells. For example, TRPP1 and TRPP2 (PKD1 and PKD2) are involved in detecting kidney urine flow (Nauli et al., 2003).

TRPA1 is named for the presence of numerous ankyrin repeats. It is activated by both natural compounds, such as thiosulfinates, cinnamaldehyde, and AITC, and artificial chemicals, such as toluene, cigarette smoke, and tear gas (Bautista et al., 2006). In addition, TRPA1 is controlled by endogenous molecules and redox conditions (Andersson et al., 2008; Bessac et al., 2008). Human TRPA1 is activated by cold and heat (Story et al., 2003; Karashima et al., 2009; Moparthi et al., 2014; Sinica et al., 2019), although its sensitivity to cold temperatures has been disputed (see Discussion). Current induction by cold stimuli alone is not as robust as agonist-induced currents, but the presence of agonists greatly attenuates cold-induced currents, suggesting an interplay between chemical and thermal sensitivities (da Costa et al., 2010; del Camino et al., 2019).

TRPA1 has both clinical and pharmaceutical significance, as it has been associated with a number of symptoms, such as itch and pain. TRPA1 is involved in histamine-independent itch caused by pruritogens, such as bile acid and chloroquine (Wilson et al., 2011; Lieu et al., 2014). The pruritic actions of cytokines require TRPA1 expressed in sensory neurons (Wilson et al., 2013; Cevikbas et al., 2014). TRPA1 mediates inflammation and scratching associated with allergic contact dermatitis; these symptoms can be mitigated by the genetic ablation of the *TRPA1* gene or the administration of the TRPA1 channel antagonists HC030031 and A-967079 (Liu et al., 2013). Itch-evoked scratching caused by atopic dermatitis is mediated by TRPA1 in dermal nerve fibers, which can be inhibited by HC030031 (Oh et al., 2013). The pain and neural damage observed in streptozotocin-induced diabetic animal models can be relieved by a derivative of HC030031 (Wei et al., 2009). Neurotoxicity, including mechanical and cold hypersensitivities resulting from chemotherapeutic agents, can be treated by blocking TRPA1 activity or deleting the *TRPA1* gene (Nassini et al., 2011; Materazzi et al., 2012; Trevisan et al., 2013). Furthermore, cold hyperalgesia upon inflammation and peripheral nerve injury have been attributed to TRPA1, whose stimulation by low temperatures is amplified markedly by agonist treatment (da Costa et al., 2010; del Camino et al., 2019). Because of the need to relieve these symptoms, TRPA1 is a frequent target of drug development. However, little success has been achieved in clinical trials (Andrade et al., 2012; Moran, 2018; Giorgi et al., 2019).

An assay with high sensitivity and high throughput is required for screening drug effectiveness. Drugs targeting ion channels can be assessed by electrophysiological measurements of cultured cells expressing the channels. Electrophysiological measurements provide precise analytical results but are generally technically difficult and time consuming. Assessing the effects on model organisms is not cost effective; additionally, TRPA1 does not have the same characteristics in both model organisms and humans (Chen and Kym, 2009; Laursen et al., 2015). For example, some trichloro(sulfanyl)ethyl benzamides serve as agonists for human TRPA1 (hTRPA1) but as antagonists for rat TRPA1 (Klionsky et al., 2007). For future clinical applications, it is critical to use hTRPA1 for functional assessments. We thus aimed to develop a bioassay using a unicellular organism expressing hTRPA1.

In this study, we used a unicellular eukaryotic organism, *Chlamydomonas reinhardtii*, to express hTRPA1. *C. reinhardtii* has been extensively used for the study of cilia and flagella and has served as a model organism for the study of ciliopathy (Keller et al., 2005; Pazour et al., 2006; Merchant et al., 2007). *C. reinhardtii* has endogenous TRP channels involved in mechanoresponses, deflagellation, mating, thermoreception, and chemoreception (Huang et al., 2007; Fujiu et al., 2011; Arias-Darraz et al., 2015; Hilton et al., 2016; Wada et al., 2020). We therefore surmised that *C. reinhardtii* may be a good model organism for expressing exogenous TRP channels. A variety of physiological responses to light, and to mechanical, thermal, and chemical stimuli have been characterized, which can be used to determine the function of TRP channels (Schmidt and Eckert, 1976; Bean, 1977; Witman, 1993; Fujiu et al., 2011; Sekiguchi et al., 2018).

In this study, we developed transgenic *C. reinhardtii* expressing hTRPA1 and found that the activity of hTRPA1 can be assessed by temperature-dependent changes in the direction of phototaxis. These changes could be inhibited by TRPA1 antagonists, including HC030031, and promoted by TRPA1 agonists, such as AITC and icilin. We propose that *C. reinhardtii* can be used in a bioassay for human TRP channel activity.

## Materials and Methods

### Cells Expressing Human TRPA1

The wild-type *C. reinhardtii* (a progeny from the mating of two wild-type strains, CC124 [mt-] and CC125 [mt+], devoid of the *agg1* mutation, Ueki et al., 2016) was used as the control and the host to express hTRPA1.

hTRPA1 cDNA (Flexi ORF clone, Promega, Madison, WI, USA) was cloned into the pChlamy 4 vector (Invitrogen, Carlsbad, CA, USA) in which the bleomycin resistance gene was replaced with the paromomycin resistance *APHVIII* gene. The construct was transformed into wild-type *C. reinhardtii* cells using an electroporator (NEPA21, Nepagene, Ichikawa, Japan). Colonies that grew on a Tris acetate phosphate (TAP) plate containing 10 µg/mL paromomycin were isolated (Gorman and Levine, 1965).

Transformants were grown in liquid TAP media for genetic analyses. Genomic DNA was isolated using the Wizard Genomic DNA purification Kit (Promega). The integration of hTRPA1 was assessed by PCR (HotStarTaq DNA polymerase, Qiagen, Hilden, Germany) using the following primers: forward: 5’-ATTTACTTATTGGTTTGGCAGTTGGC-3’ and reverse: 5’-CTAAGGCTCAAGATGGTGTGTTTTTG-3’. Primers for *C. reinhardtii* actin were used for the positive control (forward: 5’-AAGGCCAACCGCGAGAAGAT-3’ and reverse: 5’-TAATCGGTGAGGTCGCGGC-3’).

Transcription of hTRPA1 was confirmed by RT-PCR; mRNA was prepared using the TRIzol reagent and subjected to reverse transcription by SuperScript III (ThermoFisher Scientific, Waltham, MA, USA) using oligo(dT)_17_ as a primer. PCR was performed using the above polymerase and primers.

The expression of hTRPA1 was detected by western blot analysis. 100 mL of cells grown in liquid TAP media were washed with 50 mL of a buffer containing 10 mM Hepes-KOH (pH 7.4), 1 mM EGTA-KOH, and 4% sucrose. After centrifugation at 1,600 *g* for 5 min, cells were resuspended in 10 mL of 25 mM KCl, 30 mM Hepes-KOH (pH 7.4), 5 mM MgSO_4_, 1 mM DTT, 0.5 mM EGTA-KOH, and 0.1% Igepal CA-630 (MP Biomedicals, Solon, OH USA) and kept on ice for 5 min. Detergent-insoluble components were removed by centrifugation at 22,300 *g* for 30 min, and the supernatant was concentrated using an Amicon Ultra-15 cartridge (Merck Millipore, Burlington, MA, USA). After addition of 6× sample buffer with reducing agent, the samples were separated by 12.5% SDS-polyacrylamide gel electrophoresis at 250 V for 40 min (One Precast gel, Nakarai Tesque, Kyoto, Japan) and transferred onto a PVDF membrane (Immobilon-P transfer membrane, Merck Millipore). The membrane was cut between the 50 kD and 75 kD molecular marker bands. The membranes with proteins larger than 75 kD and smaller than 50 kD were used to detect hTRPA1 and aquaporin, respectively. The membranes were incubated in a blocking buffer containing 3% nonfat milk in PBST (phosphate-buffered saline with 0.5% Tween 20) for 30 min at room temperature. The blocked blots were immersed in the blocking buffer containing an anti-human TRPA1 polyclonal antibody (NB110-40763, 1:5000 dilution, Novus Biologicals, Centennial, CO, USA) or polyclonal antibodies against *C. reinhardtii* MIP1 aquaporin (AS15-2826, 1:10,000 dilution, Agrisera, Vännäs, Sweden) and incubated for 1 h. After washing 3 times (5 min each) in the blocking buffer, the blots were incubated in the blocking buffer containing the secondary antibody, Amersham ECL Rabbit IgG, HRP-linked F(ab’)_2_ fragment (from donkey, NA9340, 1:5000 dilution, GE Healthcare Life Sciences, Buckinghamshire, UK) for 1 h. The membranes were washed twice with the blocking buffer and then once with PBST. The blots were incubated in Clarity western ECL substrate (Bio-Rad, Hercules, CA, USA) prior to imaging with a gel-imaging system (ChemiDoc XRS Plus, Bio-Rad).

### Assay for Behavioral Responses

Cells were grown in minimal media (Sager and Granick, 1953) at 25°C under 12-h light/12-h dark conditions. Cells were washed twice with a solution containing 5 mM KCl, 0.3 mM CaCl_2_, 0.2 mM EGTA-KOH, and 5 mM Hepes-KOH (pH 7.4) and centrifuged at 1,600 *g* for 5 min. The cell density was measured with a cell counter (R1, Olympus, Tokyo, Japan) and adjusted to 6.0 × 10^6^ cells/mL.

The TRP channel inhibitors used in this study were 4-(3-chloro-2-pyridinyl)-N-[4-(1,1-dimethylethyl)phenyl]-1- piperazinecarboxamide (BCTC; final concentration: 20 µM), capsazepine (20 µM), HC030031 (300 µM), A-967079 (30 µM), and AP18 (100 µM). The TRP channel agonists were icilin, allyl isothiocyanate (AITC), capsaicin, and gingerol. All of the above chemicals were purchased from Fujifilm Wako Pure Chemical (Osaka, Japan) and dissolved in DMSO for the preparation of stock solutions. *Tert*-butyl hydroperoxide (*t*-BOOH) was applied to induce oxidizing conditions.

For the assessment of cellular distribution, 1 mL of the cell suspension was placed in a 1.5-mL microtube, and the microtubes were placed into an aluminum block incubator set at the desired temperature. Green- or red-light illumination was provided by an LED lamp (IP66, MEIKEE, Hong Kong) set 14.5 cm above the aluminum block. The distribution of cells was photographed after the desired time periods.

The swimming direction during phototaxis was assessed by recording the cell motility under the microscope (Matsuda et al., 1998). An aliquot of the cell suspension was placed in a trough (inside dimension: 10 mm × 10 mm × 3 mm) set on a microscope stage (IX71, Olympus). The temperature of the stage was controlled using a temperature controller (Thermo Plate, Tokai Hit, Fujinomiya, Japan). Phototactic light was applied from the side through a 500-nm bandpass filter (03FIV038, Melles Griot, Irvine, CA, USA). The trajectory of the cells was recorded using a CMOS camera (Orca-Spark, Hamamatsu Photonics, Hamamatsu, Shizuoka, Japan). Exposure time was 0.5 second per second and three sequential frames were taken 10 second after the phototactic light was turned on. The direction (θ) that the cells traveled relative to the phototactic light was obtained using ImageJ software. The phototactic index (PI) was calculated as the average of cos θ (Matsuda et al., 1998). Thus, PI approaches +1 when cells exhibit positive phototaxis and –1 when cells exhibit negative phototaxis.

## Results

### Construction of *Chlamydomonas* Cells Expressing hTRPA1

**Figure 1:**
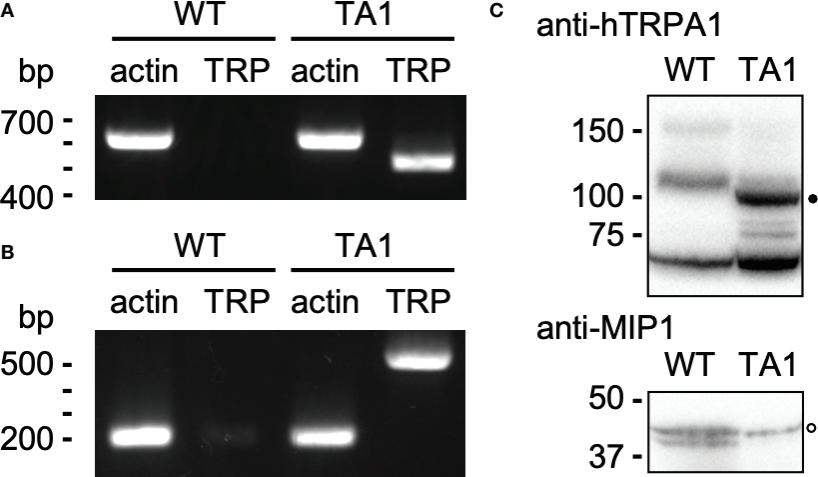
Construction of a *C. reinhardtii* transformant expressing hTRPA1. **(A)** PCR for actin and hTRPA1 using the genomic DNA from wild-type (WT) and TA1 cells as templates. The sizes expected from the genome database (https://phytozome-next.jgi.doe.gov) are 611 bp for actin and 490 bp for hTRPA1. **(B)** RT-PCR to confirm transcription. The expected sizes are 227 bp for actin and 490 bp for hTRPA1. **(C)** Western blot using anti-hTRPA1 antibody and anti-MIP1 (aquaporin) antisera. Presumed bands for hTRPA1 and MIP1 are indicated by the filled and open dots, respectively. Molecular masses are in kDa.

hTRPA1 cDNA was cloned into an expression vector for *C. reinhardtii* and transformed into wild-type cells. An isolated clone, TA1, had the gene for hTRPA1 as assessed by genomic PCR (**Figure 1A**). Actin was used as the positive control. Transcription of hTRPA1 was confirmed by RT-PCR (**Figure 1B**). The size of the actin cDNA was approximately 400 bp smaller than that of the genomic DNA because of splicing, consistent with genomics resource (https://phytozome-next.jgi.doe.gov). Antibodies against hTRPA1 detected a band at approximately 100 kDa in the detergent-soluble fraction from TA1 cells but not from wild-type cells (**Figure 1C**). This band was attributed to hTRPA1, since hTRPA1 has been reported to migrate at this molecular mass (Hatano et al., 2012). In the following experiments, TA1 was used as the strain expressing hTRPA1. Immunofluorescence microscopy was not attempted because there were several intrinsic proteins that cross-reacted with the anti-hTRPA1 antibodies.

### Phototaxis in Cells Expressing hTRPA1

**Figure 2:**
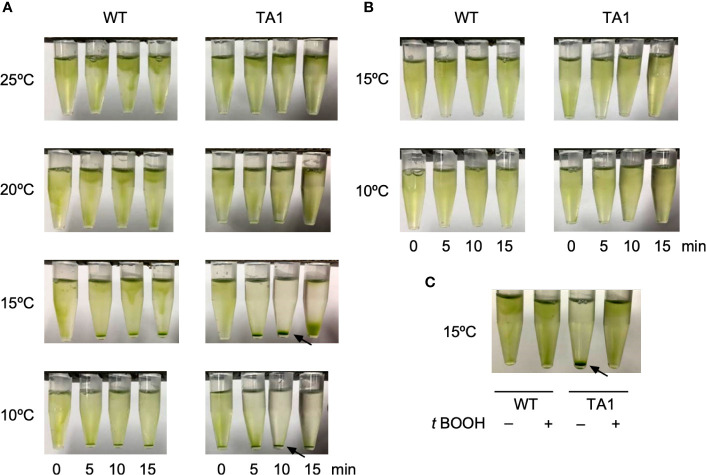
Effects of light application on the distribution of *C. reinhardtii* cells in microtubes. Wild-type (WT) cells and cells expressing hTRPA1 (TA1) were used. **(A)** Green light was applied from the top of the tube at 10°C to 25°C for 0 to 15 min. **(B)** Red light was applied from the top of the tube at 10°C or 15°C for 0 to 15 min. **(C)** Green light was applied at 15°C for 10 min in the absence or presence of 200 µM *t*-BOOH. Sedimentation of cells is indicated by the arrows.

The motility of TA1 cells was similar to that observed in wild-type cells in terms of trajectory and velocity. We examined whether the responses to chemical and thermal stimuli differed between TA1 and wild-type cells. We did not observe chemotaxis to TRPA1 agonists, such as icilin and AITC. Interestingly, we noticed that TA1 cells sedimented in a 1.5-mL microtube in an aluminum cooling block when the temperature was set to 10°C. Such a phenomenon was not evident in wild-type cells. Since the motility did not differ between TA1 and wild-type cells at any temperature, the cells probably migrated actively toward the bottom of the microtube. Possible stimuli for this directional migration are ambient light and gravity. A temperature gradient may also have existed before becoming uniform in the microtube. To discriminate between these possibilities, we applied a green light from the top of the microtube to induce phototaxis. When examined at 20°C and 25°C, both wild-type and TA1 cells migrated upward, and no sediment was present on the bottom of the tube (**Figure 2A**). At 15°C and 10°C, however, a clear accumulation of sediment was observed at the bottom of the tube containing TA1 cells, and the upper half of the microtube was almost colorless after 10 min. Faint sediment was also observed in wild-type cells, but the upper part of the tube was still green, indicating that only a portion of cells migrated downwards. The sedimentation observed in the tube containing wild-type cells was often undetectable, as will be shown below (e.g., wild-type cells at 0 µM antagonist in **Figure 4A**).

To test that the downward movement of cells at low temperature was due to green light, which induces phototaxis in *C. reinhardtii*, we instead applied red light, which does not evoke phototaxis. Total darkness was not adopted because *C. reinhardtii* cells tend to become inactive in the absence of light. The application of red light did not induce downward migration at either 10°C or 15°C (**Figure 2B**), indicating that sedimentation was caused by phototaxis rather than a response to gravity or a temperature gradient.

If the sedimentation was caused by phototaxis, then it should be affected by the intracellular redox balance, since the direction of phototaxis can be altered by changing the redox balance (Wakabayashi et al., 2011). When the redox balance was shifted to an oxidizing condition by the addition of *t*-BOOH, the sedimentation of TA1 cells at 15°C was abolished (**Figure 2C**), whereas the distribution of wild-type cells was not affected. This observation is consistent with the observation that negative phototaxis can be reversed to positive phototaxis by the addition of *t*-BOOH, supporting the idea that the sedimentation is due to negative phototaxis (Wakabayashi et al., 2011).

**Figure 3:**
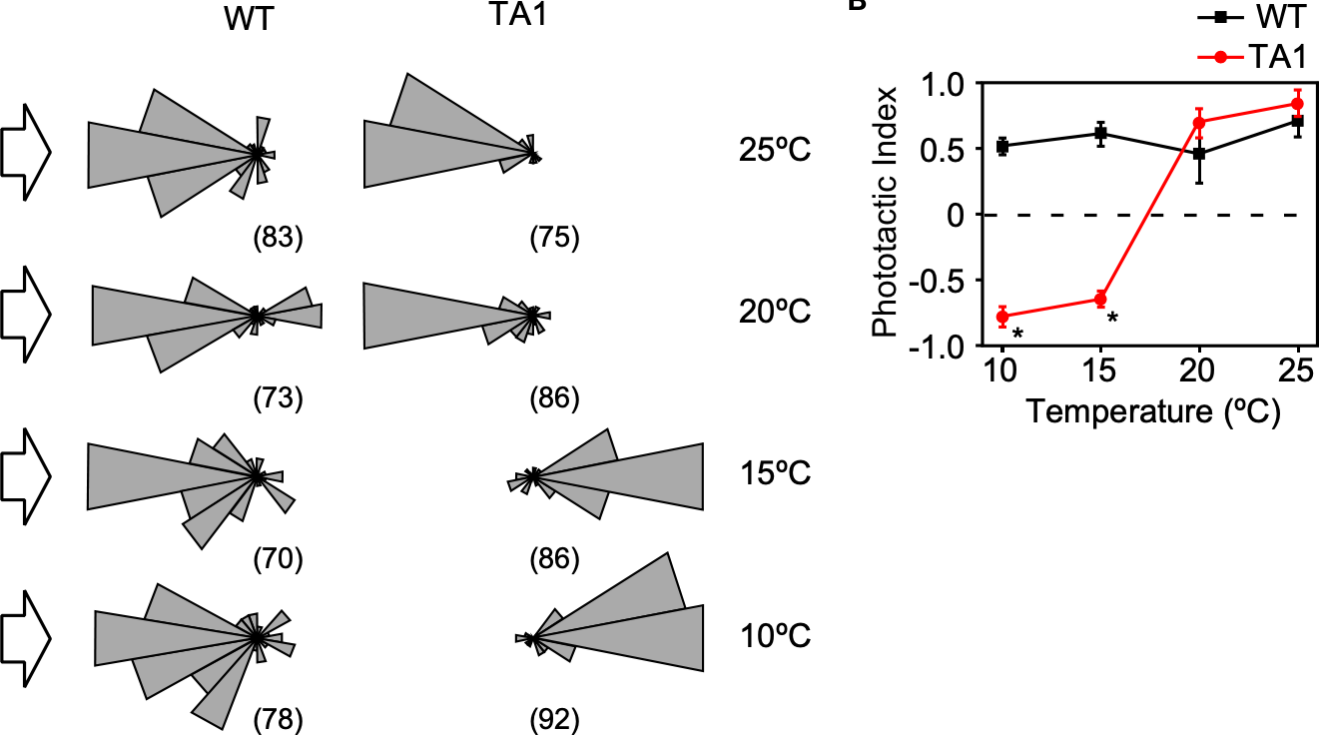
Temperature dependence of the direction of phototaxis in wild-type (WT) and TA1 cells. The light intensity was 3.6 × 10^17^ photons/m^2^/s^-1^. **(A)** A polar histogram of the direction of phototaxis when 500-nm light was applied from the left (arrow). The bin is 20 degrees. The numbers in parentheses indicate the number of cells analyzed. **(B)** Phototactic index according to temperature in WT and TA1 cells. Mean and standard deviation (n = 5). Stars indicate statistically significant difference from the corresponding value in WT (t-test, p<0.01).

To further ascertain whether TA1 cells exhibit negative phototaxis at low temperatures while wild-type cells exhibit positive phototaxis, the trajectory of swimming was recorded under the microscope. When 500-nm light was applied to one side of the trough containing wild-type cells, nearly all cells swam toward the light source at any temperature between 10°C and 25°C (**Figure 3A**). TA1 cells exhibited positive phototaxis at 20°C and 25°C, but negative phototaxis at 10°C and 15°C. The phototactic index (PI) was calculated by averaging the cosine of the trajectory of each cell. The PI, which ranges from +1 (positive phototaxis) to –1 (negative phototaxis), revealed that wild-type cells exhibited positive phototaxis from 10°C to 25°C, but that TA1 cells exhibited negative phototaxis at 10°C and 15°C, and positive phototaxis at 20°C and 25°C (**Figure 3B**).

The above observations indicate that TA1 cells exhibit negative phototaxis at 10°C and 15°C, which does not occur in wild-type cells. Given that hTRPA1 has been reported to be active at temperatures below 17°C, it is likely that the negative phototaxis exhibited by TA1 is brought about by the cold activation of hTRPA1 (Story et al., 2003) (see **Figure 7**).

### Inhibition of Negative Phototaxis by TRPA1 Antagonists

**Figure 4:**
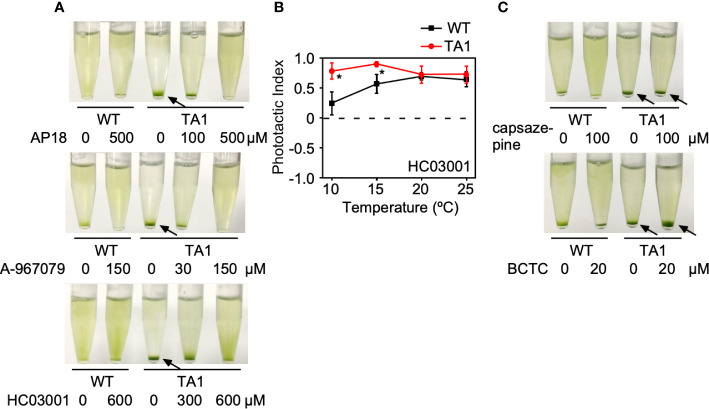
Effects of TRP channel antagonists on the phototaxis of wild-type (WT) and TA1 cells. In A and C, green light was applied from the top of the tube at 15°C for 10 min. **(A)** Cell distribution in the presence of the TRPA1 antagonists AP18, A-967079, and HC030031. **(B)** Phototactic index according to temperature in WT and TA1 cells in the presence of HC030031. Mean and standard deviation (n = 5). Stars indicate statistically significant difference from the corresponding value in WT (t-test, p<0.01). **(C)** Cell distribution in the presence of the TRPV1 antagonists capsazepine and BCTC. Sedimentation of cells is indicated by the arrows.

If the above assumption that TA1 cells exhibit negative phototaxis at low temperatures in response to hTRPA1 activation is correct, then this process should be blocked by TRPA1 antagonists. To test this hypothesis, phototaxis was assessed in the presence of inhibitors specific for TRPA1—AP18, A-967079, and HC03003. The sedimentation of TA1 cells at 15°C was weakly dispersed in the presence of 100 µM AP18 and completely dispersed in the presence of 500 µM AP18 (**Figure 4A**). The upper layer became greener as the concentration of AP18 increased. The distribution of wild-type cells was not affected. Likewise, A-967079 blocked the sedimentation of TA1 cells partially at 30 µM and totally at 150 µM. Sedimentation was not blocked at 0.01 to 10 µM (**Figure S1**). Sedimentation was inhibited also in the presence of 600 µM HC030031.

To confirm that sedimentation is blocked by a change in phototaxis, the trajectory of swimming was monitored in the presence of HC030031. When phototactic light was applied, TA1 cells exhibited positive phototaxis at all temperatures examined between 10°C and 25°C (**Figure 4B**). Positive phototaxis exhibited by wild-type cells was not affected by HC030031. This observation implies that TA1 cells exhibited positive phototaxis at low temperatures when the cold activation of TRPA1 was inhibited.

The effects of specific TRPV1 inhibitors on TA1 cell sedimentation were then examined instead of specific TRPA1 inhibitors. Neither BCTC nor capsazepine inhibited sedimentation at 15°C (**Figure 4C**).

Taken together, TRPA1 antagonists inhibit the sedimentation, or negative phototaxis, of TA1 cells at low temperatures.

### Generation of Negative Phototaxis by TRPA1 Agonists

**Figure 5:**
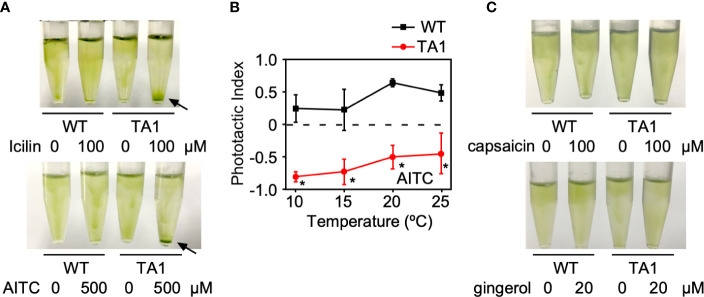
Effects of TRP channel agonists on the phototaxis of wild-type (WT) and TA1 cells. In A and C, green light was applied from the top of the tube at 25°C for 10 min. **(A)** Cell distribution in the presence of the TRPA1 agonists icilin and AITC. Sedimentation of cells is indicated by the arrows. **(B)** Phototactic index according to temperature in WT and TA1 cells in the presence of AITC. Mean and standard deviation (n = 5). Stars indicate statistically significant difference from the corresponding value in WT (t-test, p<0.01). **(C)** Cell distribution in the presence of the TRPV1 agonists capsaicin and gingerol.

If the negative and positive phototaxis by TA1 cells at low and high temperatures, respectively, is due to the presence and absence of active hTRPA1, then the activation of hTRPA1 by TRPA1 agonists would be expected to switch positive phototaxis at high temperatures to negative phototaxis. To test this idea, icilin and AITC, both TRPA1 agonists, were added to cells at 25°C. As expected, icilin and AITC induced the sedimentation of TA1 cells (**Figure 5A**). Wild-type cells did not respond to icilin or AITC, indicating that the sedimentation of TA1 cells is caused by the activity of hTRPA1.

Whether the induction of sedimentation by icilin and AITC was due to phototaxis was examined by recording the trajectory of cells when phototactic light was applied. When AITC was present, TA1 cells exhibited negative phototaxis at 20°C and 25°C, as well as at 10°C and 15°C (**Figure 5B**). Phototaxis exhibited by wild-type cells was positive in the presence of AITC at all temperatures tested.

When TRPV1 agonists, such as capsaicin and gingerol, were added instead of TRPA1 agonists at 25°C, no sedimentation was observed (**Figure 5C**).

The above observation supports the idea that the activation of hTRPA1 by TRPA1 agonists shifts the phototactic direction to negative phototaxis, even at 20°C and 25°C.

### Blockade of the Effects of TRPA1 Agonists by Antagonists

**Figure 6:**
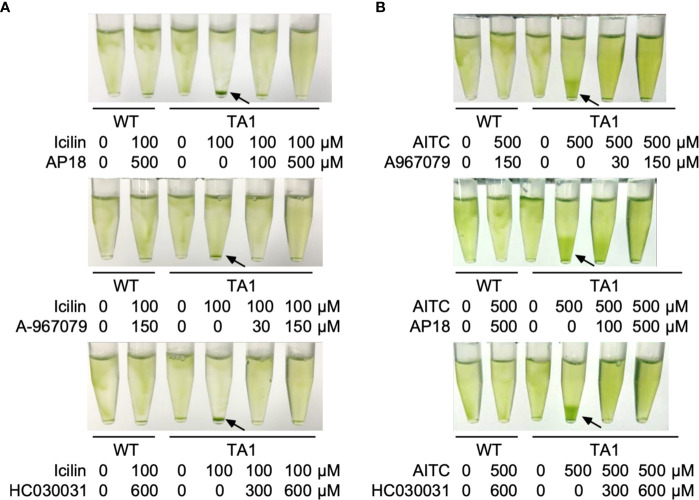
Effects of TRP channel antagonists on the action of agonists. Green light was applied from the top of the tube at 25°C for 10 min. **(A)** Effects of the TRPA1 antagonists AP18, A-967079, and HC030031 on the action of icilin. **(B)** Effects of the three TRPA1 antagonists on the action of AITC. Sedimentation of cells is indicated by the arrows.

If the sedimentation of TA1 cells by the application of TRPA1 agonists was caused by the activation of hTRPA1, then such sedimentation should be blocked by TRPA1 antagonists. This idea was tested by applying icilin or AITC in the presence of a TRPA1 inhibitor. Icilin induced the sedimentation of TA1 cells in the absence of inhibitor, but when AP18, A-967079, or HC030031 was present, the sedimentation was much weaker (**Figure 6A**). The distribution of wild-type cells was not affected by the simultaneous application of icilin and a TRPA1 inhibitor. Similarly, sedimentation induced by AITC was blocked by TRPA1 antagonists (**Figure 6B**).

## Discussion

**Figure 7:**
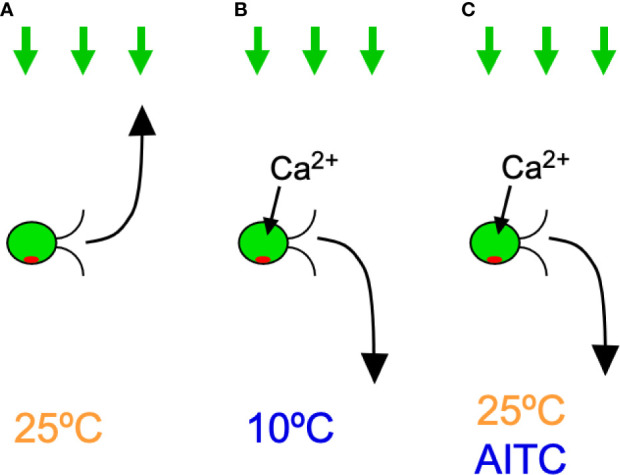
Summary of the results in *C. reinhardtii* cells expressing hTRPA1. Green arrows on the top indicate light for phototaxis and the curved arrows in front of cells indicate phototactic turn. Ca^2+^ influx through hTRPA1 is also indicated. **(A)** Positive phototaxis at 25°C. **(B)** Negative phototaxis at 10°C. **(C)** Negative phototaxis in the presence of AITC at 25°C.

This study showed that *C. reinhardtii* cells expressing hTRPA1 exhibit negative phototaxis at temperatures of 15°C or lower (**Figure 7**). This response is likely due to the cold activation of hTRPA1 because negative phototaxis was not observed in wild-type cells, negative phototaxis was blocked by TRPA1 inhibitors, and the temperature range coincided with that necessary for hTRPA1 activation (≤17°C, Story et al., 2003). Negative phototaxis can also be induced by agonists for hTRPA1, even at temperatures of 20°C or higher. This response also appears to be due to hTRPA1 because wild-type cells did not respond to hTRPA1 agonists and the response was blocked by hTRPA1 antagonists. Taken together, transgenic *C. reinhardtii* cells have acquired sensitivity to TRPA1 agonists, antagonists, and low temperatures.

Since TA1 cells are sensitive to TRPA1 agonists and antagonists, the phototaxis assay shows promise for assessing whether a given substance is an agonist or antagonist for TRPA1. A substance can be judged to be an antagonist if TA1 cells exhibit positive phototaxis at low temperatures. Conversely, a substance can be judged to be an agonist if TA1 cells exhibit negative phototaxis at high temperatures. Such responses are evident after 10 min, and the maintenance of *C. reinhardtii* cells is cost efficient. Qualitative data can be obtained by analyzing the trajectory of swimming during phototaxis, and several software programs exist that can automatically analyze the trajectories of individual cells. The primary limitation of this assay is that while it can be used to evaluate the functions of agonists and antagonists at a cellular level, the signal transduction pathway for the determination of phototaxis direction is still to be elucidated. Another limitation is that the antagonist concentration needed to block negative phototaxis is high compared to the IC_50_ determined electrophysiologically. For example, A-967079 inhibited negative phototaxis negligibly at 10 µM, partially at 30 µM, and totally at 100 µM, suggesting IC_50_ in the present assay is in the order of 10 µM whereas IC_50_ determined electrophysiologically is 67 nM (McGaraughty et al., 2010). We surmise high concentration is needed because of the presence of cell wall in *C. reinhardtii* or because a reminiscent activity of TRPA1 is enough to evoke fractional directional movement resulting in net migration.

The mechanism by which the direction of phototaxis is determined in *C. reinhardtii* is not fully understood. The phototactic turn during phototaxis is probably brought about by changing the balance of the propulsive force between the two flagella, which is controlled by submicromolar levels of Ca^2+^ (Kamiya and Witman, 1984). Given that hTRPA1 is permeable to cations, including Ca^2+^, it is probable that the elevation of Ca^2+^ concentration through hTRPA1 induces negative phototaxis (Story et al., 2003) (**Figure 7**). In support of this idea, an increase in intracellular Ca^2+^ concentration through application of Ca^2+^ permeable ionophores results in rapid phototactic migration to the bottom of a tube in *C. reinhardtii* (Pasquale and Goodenough, 1987). Ca^2+^ may control the phosphorylation status of the photoreceptor, channelrhodopsin (ChR) 1, and the direction of phototaxis (Böhm et al., 2019). The direction of phototaxis is also under the influence of the redox state. Positive phototaxis occurs when the intracellular redox condition is changed to oxidizing conditions, and negative phototaxis occurs under reducing conditions (Wakabayashi et al., 2011). It is unlikely that TRPA1 changes the redox status, even though it is sensitive to redox status (Takahashi et al., 2018). Some reports have shown that negative phototaxis can occur by a loss of *AGG1* gene or a defect in the eyespot carotenoid layer in *C. reinhardtii* (Ide et al., 2016; Ueki et al., 2016). There is, however, no data to conclusively correlate the activity of TRPA1 with AGG1 levels or eyespot carotenoid defects.

When considering whether hTRPA1 is thermosensitive, it is intriguing to note that hTRPA1 is activated by cold when expressed in *C. reinhardtii* cells. hTRPA1 was first reported to be activated by cold (Story et al., 2003), but a following study failed to confirm its cold activation (Jordt et al., 2004). TRPA1 was later found to be activated by Ca^2+^, and a cold-induced increase in Ca^2+^ levels might be responsible for cold-activation of hTRPA1 (Zurborg et al., 2007). The insensitivity of hTRPA1 to cold was considered a species difference, since rat TRPA1 and mouse TRPA1 were activated by cold but hTRPA1 was not (Chen and Kym, 2009). Despite these findings, when reconstituted into the lipid bilayer, hTRPA1 was found to be activated by both cold and heat (Moparthi et al., 2016). Its cold sensitivity was under the control of the redox condition. Furthermore, a detailed analysis of hTRPA1 reconstituted into the lipid bilayer revealed that the voltage dependence of hTRPA1 is modulated by temperature (Sinica et al., 2019). Taken together, we believe that hTRPA1 is intrinsically sensitive to temperature, which is affected greatly by cellular conditions, including Ca^2+^ levels and redox conditions, although we admit that further study is required to confirm this hypothesis.

Low motility is also possible to contribute to the sedimentation of cells at low temperatures. Recently, we found that capsaicin and gingerol induced deflagellation and blocked motility in *C. reinhardtii* (Wada et al., 2020). Our failure to observe sedimentation when capsaicin or gingerol was applied (**Figure 5C**) was probably due to a low sedimentation speed. Since the sedimentation speed of immobilized *C. reinhardtii* cells is approximately 2 µm/s, cells would sediment only 1.2 mm after 10 min (Yoshimura et al., 2003), indicating that passive sedimentation is not sufficient to produce an apparent sediment in a 27-mm-deep solution in a microtube. The swimming velocity, on the other hand, is much faster: approximately 40 µm/s at 10°C and 110 µm/s at 25°C (Sekiguchi et al., 2018). Cells would migrate 48 mm in 10 min at 10°C, which is greater than the depth of the solution. This estimation indicates that the observed sediment is due to active downward swimming rather than passive sedimentation or impaired motility.

To conclude, this study showed that *C. reinhardtii* expressing hTRPA1 exhibits a clear response to TRPA1 agonists and antagonists. The assay described here can be used to study other clinically important TRP channels. *C. reinhardtii* has long been used in basic research on ciliary motility and photosynthesis, but applied research using *C. reinhardtii* for biofuel and bioproduct production is growing rapidly (Scranton et al., 2015). *C. reinhardtii* also shows promise for its use in bioassays to evaluate the functions of exogenous proteins.

## Data Availability Statement

The raw data supporting the conclusions of this article will be made available by the authors, without undue reservation.

## Author Contributions

MY, RY, and YS performed the research and analyzed data. KY designed the research and wrote the paper.

## Conflict of Interest

The authors declare that the research was conducted in the absence of any commercial or financial relationships that could be construed as a potential conflict of interest.

## References

[B1] Andersson D. A. Gentry C. Moss S. Bevan S. (2008). Transient receptor potential A1 is a sensory receptor for multiple products of oxidative stress. J. Neurosci. 28 (10), 2485–2494. 10.1523/JNEUROSCI.5369-07.2008 18322093 PMC2709206

[B2] Andrade E. L. Meotti F. C. Calixto J. B. (2012). TRPA1 antagonists as potential analgesic drugs. Pharmacol. Ther. 133 (2), 189–204. 10.1016/j.pharmthera.2011.10.008 22119554

[B3] Arias-Darraz L. Cabezas D. Colenso C. K. Alegría-Arcos M. Bravo-Moraga F. Varas-Concha I. (2015). A transient receptor potential ion channel in *Chlamydomonas* shares key features with sensory transduction-associated TRP channels in mammals. Plant Cell 27 (1), 177–188. 10.1105/tpc.114.131862 25595824 PMC4330573

[B4] Bandell M. Story G. M. Hwang S. W. Viswanath V. Eid S. R. Petrus M. J. (2004). Noxious cold ion channel TRPA1 is activated by pungent compounds and bradykinin. Neuron 41 (6), 849–857. 10.1016/s0896-6273(04)00150-3 15046718

[B5] Bautista D. M. Jordt S. E. Nikai T. Tsuruda P. R. Read A. J. Poblete J. (2006). TRPA1 mediates the inflammatory actions of environmental irritants and proalgesic agents. Cell 124 (2), 1269–1282. 10.1016/j.cell.2006.02.023 16564016

[B6] Bean B. (1977). Geotactic behavior of *Chlamydomonas* . J. Protozool. 24 (3), 394– 401. 10.1111/j.1550-7408.1977.tb04759.x| 915843

[B7] Bessac B. F. Sivula M. von Hehn C. A. Escalera J. Cohn L. Jordt S. E. (2008). TRPA1 is a major oxidant sensor in murine airway sensory neurons. J. Clin. Invest. 118 (5), 1899–1910. 10.1172/JCI34192 18398506 PMC2289796

[B8] Böhm M. Boness D. Fantisch E. Erhard H. Frauenholz J. Kowalzyk Z. (2019). Channelrhodopsin-1 Phosphorylation Changes with Phototactic Behavior and Responds to Physiological Stimuli in *Chlamydomonas* . Plant Cell 31 (4), 886–910. 10.1105/tpc.18.00936 30862615 PMC6501600

[B9] Caterina M. J. Schumacher M. A. Tominaga M. Rosen T. A. Levine J. D. Julius D. (1997). The capsaicin receptor: a heat-activated ion channel in the pain pathway. Nature 389 (6653), 816–824. 10.1038/39807 9349813

[B10] Cevikbas F. Wang X. Akiyama T. Kempkes C. Savinko T. Antal A. (2014). A sensory neuron-expressed IL-31 receptor mediates T helper cell-dependent itch: Involvement of TRPV1 and TRPA1. J. Allergy Clin. Immunol. 133 (2), 448–460. 10.1016/j.jaci.2013.10.048 24373353 PMC3960328

[B11] Chen J. Kym P. R. (2009). TRPA1: the species difference. J. Gen. Physiol. 133 (6), 623–625. 10.1085/jgp.200910246 19468076 PMC2713145

[B12] Clapham D. E. (2003). TRP channels as cellular sensors. Nature 426 (6966), 517–524. 10.1038/nature02196 14654832

[B13] da Costa D. S. Meotti F. C. Andrade E. L. Leal P. C. Motta E. M. Calixto J. B. (2010). The involvement of the transient receptor potential A1 (TRPA1) in the maintenance of mechanical and cold hyperalgesia in persistent inflammation. Pain 148 (3), 431–437. 10.1016/j.pain.2009.12.002 20056530

[B14] del Camino D. Murphy S. Heiry M. Barrett L. B. Earley T. J. Cook (2019). TRPA1 contributes to cold hypersensitivity. J. Neurosci. 30 (45), 15165–15174. 10.1523/JNEUROSCI.2580-10.2010 PMC3021322 21068322

[B15] Fujiu K. Nakayama Y. Iida H. Sokabe M. Yoshimura K. (2011). Mechanoreception in motile flagella of *Chlamydomonas* . Nat. Cell Biol. 13 (5), 630–632. 10.1038/ncb2214 21478860

[B16] Giorgi S. Nikolaeva-Koleva M. Alarcón-Alarcón D. Butrón L. González-Rodríguez S. (2019). Is TRPA1 burning down TRPV1 as druggable target for the treatment of chronic pain? Int. J. Mol. Sci. 20 (12):2906. 10.3390/ijms20122906 PMC6627658 31197115

[B17] Gorman D. S. Levine R. P. (1965). Cytochrome f and plastocyanin: their sequence in the photosynthetic electron transport chain of *Chlamydomonas reinhardi* . Proc. Natl. Acad. Sci. U. S. A. 54 (6), 1665–1669. 10.1073/pnas.54.6.1665 4379719 PMC300531

[B18] Hatano N. Itoh Y. Suzuki H. Muraki Y. Hayashi H. Onozaki K. (2012). Hypoxia-inducible factor-1α (HIF1α) switches on transient receptor potential ankyrin repeat 1 (TRPA1) gene expression via a hypoxia response element-like motif to modulate cytokine release. J. Biol. Chem. 287 (38), 31962–31972. 10.1074/jbc.M112.361139 22843691 PMC3442528

[B19] Hilton L. K. Meili F. Buckoll P. D. Rodriguez-Pike J. C. Choutka C. P. Kirschner J. A. (2016). A forward genetic screen and whole genome sequencing identify deflagellation defective mutants in *Chlamydomonas*, including assignment of ADF1 as a TRP channel. G3 (Bethesda) 6 (10), 3409–3418. 10.1534/g3.116.034264 27520959 PMC5068960

[B20] Huang K. Diener D. R. Mitchell A. Pazour G. J. Witman G. B. Rosenbaum J. L. (2007). Function and dynamics of PKD2 in *Chlamydomonas reinhardtii* flagella. J. Cell. Biol. 179 (3), 501–514. 10.1083/jcb.200704069 17984324 PMC2064795

[B21] Ide T. Mochiji S. Ueki N. Yamaguchi K. Shigenobu S. Hirono M. (2016). Identification of the agg1 mutation responsible for negative phototaxis in a “wild-type” strain of. Chlamydomonas reinhardtii. Biochem. Biophys. Rep. 7, 379–385. 10.1016/j.bbrep.2016.07.016 28955929 PMC5613634

[B22] Jordt S. E. Bautista D. M. Chuang H. H. McKemy D. D. Zygmunt P. M. Högestätt E. D. (2004). Mustard oils and cannabinoids excite sensory nerve fibres through the TRP channel ANKTM1. Nature 427 (6971), 260–265. 10.1038/nature02282 14712238

[B23] Kamiya R. Witman G. B. (1984). Submicromolar levels of calcium control the balance of beating between the two flagella in demembranated models of *Chlamydomonas* . J. Cell Biol. 98, 97–107. 10.1083/jcb.98.1.97 6707098 PMC2112995

[B24] Karashima Y. Talavera K. Everaerts W. Janssens A. Kwan K. Y. Vennekens R. (2009). TRPA1 acts as a cold sensor in vitro and in vivo. Proc. Natl. Acad. Sci. U. S. A. 106 (4), 1273–1278. 10.1073/pnas.0808487106 19144922 PMC2633575

[B25] Keller L. C. Romijn E. P. Zamora I. Yates J. R. 3rd. Marshall W. F. (2005). Proteomic analysis of isolated *Chlamydomonas* centrioles reveals orthologs of ciliary-disease genes., (2005). Curr. Biol. 15 (12), 1090–1098. 10.1016/j.cub.2005.05.024 15964273

[B26] Klionsky L. Tamir R. Gao B. Wang W. Immke D. C. Nishimura (2007). Species-specific pharmacology of Trichloro(sulfanyl)ethyl benzamides as transient receptor potential ankyrin 1 (TRPA1) antagonists. Mol. Pain 3:39. 10.1186/1744-8069-3-39 18086308 PMC2222611

[B27] Laursen W. J. Anderson E. O. Hoffstaetter L. J. Bagriantsev S. N. Gracheva E. O. (2015). Species-specific temperature sensitivity of TRPA1. Temp. (Austin) 2 (2), 214–226. 10.1080/23328940.2014.1000702 PMC4843866 27227025

[B28] Lieu T. Jayaweera G. Zhao P. Poole D. P. Jensen D. Grace M. (2014). The bile acid receptor TGR5 activates the TRPA1 channel to induce itch in mice. Gastroenterology 147 (6), 1417–1428. 10.1053/j.gastro.2014.08.042 25194674 PMC4821165

[B29] Liu B. Escalera J. Balakrishna S. Fan L. Caceres A. II Robinson E. (2013). TRPA1 controls inflammation and pruritogen responses in allergic contact dermatitis. FASEB J. 27 (9), 3549–3563. 10.1096/fj.13-229948 23722916 PMC3752543

[B30] Materazzi S. Fusi C. Benemei S. Pedretti P. Patacchini R. Nilius B. (2012). TRPA1 and TRPV4 mediate paclitaxel-induced peripheral neuropathy in mice via a glutathione-sensitive mechanism. Pflügers Arch. 463 (4), 561–569. 10.1007/s00424-011-1071-x 22258694

[B31] Matsuda A. Yoshimura K. Sineshchekov O. A. Hirono M. Kamiya R. (1998). Isolation and characterization of novel *Chlamydomonas* mutants that display phototaxis but not photophobic response. Cell Motil. Cytoskel. 41 (4), 353–362. 10.1002/(SICI)1097-0169(1998)41:4<353::AID-CM7>3.0.CO;2-0| 9858159

[B32] McGaraughty S. Chu K. L. Perner R. J. Didomenico S. Kort M. E. Kym P. R. (2010). TRPA1 modulation of spontaneous and mechanically evoked firing of spinal neurons in uninjured, osteoarthritic, and inflamed rats. Mol. Pain 6 (1), 14. 10.1186/1744-8069-6-14 20205719 PMC2841076

[B33] Merchant S. S. Prochnik S. E. Vallon O. Harris E. H. Karpowicz S. J. Witman G. B. (2007). The *Chlamydomonas* genome reveals the evolution of key animal and plant functions. Science 318 (5848), 245–250. 10.1126/science.1143609 17932292 PMC2875087

[B34] Moparthi L. Survery S. Kreir M. Simonsen C. Kjellbom P. Högestätt E. D. (2014). Human TRPA1 is intrinsically cold- and chemosensitive with and without its N-terminal ankyrin repeat domain. Proc. Natl. Acad. Sci. U. S. A. 111 (47), 16901–16906. 10.1073/pnas.1412689111 25389312 PMC4250169

[B35] Moparthi L. Kichko T. I. Eberhardt M. Högestätt E. D. Kjellbom P. Johanson U. (2016). Human TRPA1 is a heat sensor displaying intrinsic U-shaped thermosensitivity. Sci. Rep. 6, 28763. 10.1038/srep28763 27349477 PMC4923899

[B36] Moran M. M. (2018). TRP Channels as Potential Drug Targets. Annu. Rev. Pharmacol. Toxicol. 58, 309–330. 10.1146/annurev-pharmtox-010617-052832 28945977

[B37] Nassini R. Gees M. Harrison S. De Siena G. Materazzi S. Moretto N. (2011). Oxaliplatin elicits mechanical and cold allodynia in rodents via TRPA1 receptor stimulation. Pain 152 (7), 1621–1631. 10.1016/j.pain.2011.02.051 21481532

[B38] Nauli S. M. Alenghat F. J. Luo Y. Williams E. Vassilev P. Li X. (2003). Polycystins 1 and 2 mediate mechanosensation in the primary cilium of kidney cells. Nat. Genet. 33 (2), 129–137. 10.1038/ng1076 12514735

[B39] Oh M. H. Oh S. Y. Lu J. Lou H. Myers A. Zhu Z. (2013). TRPA1-dependent pruritus in IL-13-induced chronic atopic dermatitis. J. Immunol. 191 (11), 5371–5382. 10.4049/jimmunol.1300300 24140646 PMC4175413

[B40] Pasquale S. M. Goodenough U. W. (1987). Cyclic AMP functions as a primary sexual signal in gametes of *Chlamydomonas reinhardtii* . J. Cell Biol. 105 (5), 2279–2292. 10.1083/jcb.105.5.2279 2824527 PMC2114871

[B41] Pazour G. J. Agrin N. Walker B. L. Witman G. B. (2006). Identification of predicted human outer dynein arm genes: candidates for primary ciliary dyskinesia genes. J. Med. Genet. 43 (1), 62–73. 10.1136/jmg.2005.033001 15937072 PMC2593024

[B42] Sager R. Granick S. (1953). Nutritional studies with *Chlamydomonas reinhardi* . Ann N. Y. Acad. Sci. 56 (5), 831–838. 10.1111/j.1749-6632.1953.tb30261.x 13139273

[B43] Schmidt J. A. Eckert R. (1976). Calcium couples flagellar reversal to photostimulation in *Chlamydomonas reinhardtii* . Nature 262 (5570), 713–715. 10.1038/262713a0 958445

[B44] Scranton M. A. Ostrand J. T. Fields F. J. Mayfield S. P. (2015). *Chlamydomonas* as a model for biofuels and bio-products production. Plant J. 82 (3), 523–531. 10.1111/tpj.12780 25641390 PMC5531182

[B45] Sekiguchi M. Kameda S. Kurosawa S. Yoshida M. Yoshimura K. (2018). Thermotaxis in *Chlamydomonas* is brought about by membrane excitation and controlled by redox conditions. Sci. Rep. 8 (1), 16114. 10.1038/s41598-018-34487-4 30382191 PMC6208428

[B46] Sinica V. Zimova L. Barvikova K. Macikova L. Barvik I. Vlachova V. (2019). Human and mouse TRPA1 are heat and cold sensors differentially tuned by voltage. Cells 9 (1), 57. 10.3390/cells9010057 PMC7016720 31878344

[B47] Story G. M. Peier A. M. Reeve A. J. Eid S. R. Mosbacher J. Hricik T. R. (2003). ANKTM1, a TRP-like channel expressed in nociceptive neurons, is activated by cold temperatures. Cell 112 (6), 819–829. 10.1016/s0092-8674(03)00158-2 12654248

[B48] Takahashi N. Chen H. Y. Harris I. S. Stover D. G. Selfors L. M. Bronson R. T. (2018). Cancer cells co-opt the neuronal redox-sensing channel TRPA1 to promote oxidative-stress tolerance. Cancer Cell 33 (6), 985–1003.e7. 10.1016/j.ccell.2018.05.001 29805077 PMC6100788

[B49] Trevisan G. Materazzi S. Fusi C. Altomare A. Aldini G. Lodovici M. (2013). Novel therapeutic strategy to prevent chemotherapy-induced persistent sensory neuropathy by TRPA1 blockade. Cancer Res. 73 (10), 3120–3131. 10.1158/0008-5472.CAN-12-4370 23477783

[B50] Ueki N. Ide T. Mochiji S. Kobayashi Y. Tokutsu R. Ohnishi N. (2016). Eyespot-dependent determination of the phototactic sign in *Chlamydomonas reinhardtii* . Proc. Natl. Acad. Sci. U. S. A. 113 (19), 5299–5304. 10.1073/pnas.1525538113 27122315 PMC4868408

[B51] Wada M. Kaizuka I. Yoshimura K. (2020). Responses to transient receptor potential (TRP) channel agonists in *Chlamydomonas reinhardtii* . Biol. Open 9, bio053140. 10.1242/bio.053140 32641289 PMC7358129

[B52] Wakabayashi K. Misawa Y. Mochiji S. Kamiya R. (2011). Reduction-oxidation poise regulates the sign of phototaxis in *Chlamydomonas reinhardtii* . Proc. Natl. Acad. Sci. U.S.A. 108 (27), 11280–11284. 10.1073/pnas.1100592108 21690384 PMC3131381

[B53] Wei H. Hämäläinen M. M. Saarnilehto M. Koivisto A. Pertovaara A. (2009). Attenuation of mechanical hypersensitivity by an antagonist of the TRPA1 ion channel in diabetic animals. Anesthesiology 111 (1), 147–154. 10.1097/ALN.0b013e3181a1642b 19512877

[B54] Wilson S. R. Gerhold K. A. Bifolck-Fisher A. Liu Q. Patel K. N. Dong X. (2011). TRPA1 is required for histamine-independent, Mas-related G protein-coupled receptor-mediated itch. Nat. Neurosci. 14 (5), 595–602. 10.1038/nn.2789 21460831 PMC3181150

[B55] Wilson S. R. Thé L. Batia L. M. Beattie K. Katibah G. E. McClain S. P. (2013). The epithelial cell-derived atopic dermatitis cytokine TSLP activates neurons to induce itch. Cell 155 (2), 285–295. 10.1016/j.cell.2013.08.057 24094650 PMC4041105

[B56] Witman G. B. (1993). *Chlamydomonas* phototaxis. Trends Cell Biol. 3 (11), 403–408. 10.1016/0962-8924(93)90091-e 14731659

[B57] Yoshimura K. Matsuo Y. Kamiya R. (2003). Gravitaxis in *Chlamydomonas reinhardtii* studied with novel mutants. Plant Cell Physiol. 44 (10), 1112–1118. 10.1093/pcp/pcg134 14581636

[B58] Zurborg S. Yurgionas B. Jira J. A. Caspani O. Heppenstall P. A. (2007). Direct activation of the ion channel TRPA1 by Ca^2+^ . Nat. Neurosci. 10 (3), 277–279. 10.1038/nn1843 17259981

