# Supplementary material for: Transgenic Chlamydomonas Expressing Human Transient Receptor Potential Ankyrin 1 (TRPA1) Channels to Assess the Effect of Agonists and Antagonists
Source: Front Pharmacol. 2020 Sep 29;11:578955. doi: 10.3389/fphar.2020.578955 (PMC7550780; doi:10.3389/fphar.2020.578955)
Supplement: Supplementary file 1 [file DataSheet_1.pdf]

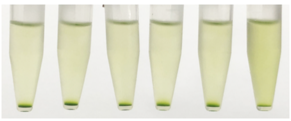

Figure S1. Effect of low concentrations of A-967079 on the phototaxis in TA1 cells. Green light was applied at the top of the tube at 15ºC for 10 min.
